# How Memory Shapes Second-Language Vocabulary Acquisition in Primary School Learners

**DOI:** 10.3390/jintelligence14060092

**Published:** 2026-06-01

**Authors:** Caterina Artuso, Loreta Cannito, Eugenio Trotta, Paola Palladino

**Affiliations:** 1 Department of Education Sciences, University of Genova, 16126 Genova, Italy; caterina.artuso@unige.it; 2 Department of Social Sciences, University of Foggia, 71121 Foggia, Italy; 3 Department of Human Studies, University of Foggia, 71121 Foggia, Italy; eugenio.trotta@unifg.it (E.T.); paola.palladino@unifg.it (P.P.)

**Keywords:** vocabulary learning, second language, gender effects, memory systems

## Abstract

This longitudinal study investigates the interplay between memory systems and second-language (L2) vocabulary acquisition in primary school children (N = 82, second and third graders). While current models emphasize the phonological loop, the specific link between short- and long-term memory remains under-explored. Over a six-month period, we assessed short-term memory and long-term memory predictors of L2 growth. The results indicate that delayed non-word recall, rather than immediate repetition, is the primary predictor of vocabulary acquisition. Furthermore, a significant gender–memory interaction was observed, as females initially outperformed males; however, this advantage diminished over time. These findings suggest that initial long-term consolidation—as also suggested by a notable primacy effect—may represent a critical missing link in L2 acquisition models and that cognitive strategies for word learning may diverge by gender.

## 1. Introduction

The acquisition of second-language (L2) vocabulary is not merely a linguistic milestone but a fundamental pillar of educational equity and social integration in increasingly multilingual classrooms (Vertovec, 2023). Within the context of early schooling, L2 proficiency acts as a critical academic performance predictor (Jiang et al., 2025); a restricted vocabulary at this developmental stage is a primary predictor of low reading proficiency (Tong et al., 2023), thus contributing to a widening achievement gap between native and non-native peers. Theoretically, investigating L2 development during these formative years is essential to refining models of bilingual cognition, as children must navigate the dual challenge of conceptual mapping and linguistic labeling during a window of significant neuroplasticity (Faitaki et al., 2021). Consequently, understanding the cognitive architecture of word retention is not an isolated psycholinguistic exercise, but also a necessary step toward designing inclusive pedagogical frameworks that mitigate systemic educational inequality (Castro & Prishker, 2019).

Following the literature, which highlights the primary role of phonological passive memory storage on first/second-language (L2) vocabulary learning (mainly assessed via immediate non-word repetition tasks, e.g., Gathercole et al., 1992, 1997; Palladino & Ferrari, 2008; Karousou & Nerantzaki, 2022; Gagné et al., 2025), this study aimed to further investigate how different memory systems influence L2 learning. Specifically, through a longitudinal study (two timepoints over six months), we administered several tasks to measure verbal short-term memory (forward and backward span), phonological short-term memory (immediate non-word recall), and long-term memory (delayed non-word recall) to determine which measures are better predictors of L2 vocabulary acquisition.

To date, to our knowledge, no study has investigated all these recall components beyond immediate non-word repetition or phonological sensitivity (e.g., Gathercole, 2006; Morra & Camba, 2009). Indeed, they focused on long-term memory, referred to as long-term knowledge, rather than long-term recall. Therefore, the present study aimed to explore whether long-term memory (delayed non-word recall) plays a predictive role in L2 vocabulary learning. In our view, the initial stages of long-term consolidation, measured through the delayed non-word recall task, may significantly contribute to explaining the role of different memory components in L2 learning.

While the short-term component should allow for the temporary maintenance of verbal information, actual lexical learning requires these traces to be transferred into long-term memory systems. In this study, we argue that delayed recall is not merely a memory test, but a proxy for the consolidation process necessary to anchor new L2 terms into the learner’s mental lexicon.

To this end, we administered a delayed non-word recall targeting long-term memory, a methodology successfully adopted by Bishop et al. (2012) in children with typical development and with language impairments.

A clear conceptual distinction should be maintained between long-term memory operationalized as accumulated lexical–semantic knowledge (e.g., vocabulary) and long-term memory operationalized as the delayed recall of newly encoded information. Vocabulary knowledge reflects the breadth and depth of crystallized semantic representations acquired through prolonged learning and repeated exposure and is therefore typically considered an index of semantic memory. In contrast, delayed recall tasks assess the retention and retrieval of episodically encoded material after a temporal delay, thus primarily indexing episodic memory processes, including consolidation and retrieval efficiency. Whereas vocabulary measures capture stable, experience-dependent knowledge structures, delayed recall paradigms evaluate the integrity of memory for specific learning episodes.

First, we examined the development of L2 learning, expecting an improvement in performance as age increases. Indeed, different predictors can be found at different levels of L2 vocabulary knowledge, as the increasing role of previous vocabulary knowledge has already been shown (e.g., Morra & Camba, 2009; Gathercole, 2006). Further, we exploratorily examined the development of L2 learning with respect to gender. While some studies agree that gender does not have a significant impact on learning languages (e.g., Kheder & Rouabhia, 2023; Taguchi, 2002), others hold different views, and reported that motivation and learning style, among others, could generate differences between females and males with respect to learning languages. For example, females appear to gain more than males on various measures of language performance such as verbal accuracy and general English mastery tests (Kheder & Rouabhia, 2023). Females prove to be better in terms of vocabulary strength, verbal fluency, and quality of speech; on the other hand, when it comes to writing skills, males are better (Tatarinceva, 2009). However, to our knowledge, no specific studies in the literature have investigated how different memory systems and gender may interact in second-language learning.

Based on existing studies in the literature, we expected females to show an advantage in the timing of L2 acquisition. Indeed, previous studies suggest an earlier developmental trajectory in females compared to males. Brouwer et al. (2021), in a longitudinal study of participants aged 9–23 years, assessed brain age differences between males and females. On average, females were found to be ahead of males by up to one year, although both genders showed similar overall developmental trajectories. Thus, while developmental patterns were comparable, females tended to progress through them earlier.

After assessing the relationship between L2 learning and memory systems (i.e., span, immediate recall, and delayed non-word recall) at both timepoints, we investigated which memory measures best predict L2 learning, anticipating that delayed non-word recall would play a particularly prominent role. This task, which is usually not used to assess how memory affects L2 outcomes, may be considered an indicator of long-term memory consolidation (Bishop et al., 2012).

In addition, to further an in-depth investigation of the delayed recall performance, we conducted an analysis that focused on specific recall effects. Indeed, we anticipated a possible primacy effect on delayed non-word memory recall. If this is the case, then we might hypothesize that this delayed recall produces an initial long-term consolidation (see, e.g., seminal studies on primacy/recency memory effects, Baddeley, 1970; Page & Norris, 1998). Hence, our aim was to investigate the interplay between memory systems and L2 vocabulary acquisition in Italian primary school children. The rest of the paper is organized as follows. Section 2 provides a theoretical overview of the project. Section 3, Section 4, Section 5 and Section 6 deal with the methodology, followed by the results, the discussion, and the conclusion.

## 2. Theoretical Background

Learning vocabulary is the basis for building language knowledge. Many studies, indeed, focused on vocabulary learning and the underlying mechanisms that may account for the acquisition of new words. Following Baddeley’s working memory (WM) model (Baddeley & Hitch, 1974), short-term verbal/phonological memory is a crucial component in learning vocabulary (see, for example, Baddeley et al., 1998; Gathercole & Baddeley, 1989). However, in the process of learning, we also experience the learning of new words in a different language, a foreign or second language (for the aims of the present paper, the expressions “foreign language” and “second language” will be used as synonyms), that may require additional or different memory resources to be involved.

When a new language is learned at school, children already possess a good theory of mind; a second language, compared to a native language, requires primarily a new phonological code imposed on abstract knowledge when learning L2 (Bernhardt, 2000). During the learning of a second language (L2), on the one hand, native language (L1) promotes the acquisition of a L2 via mechanisms of assimilation and generalization. On the other hand, native language hampers L2 learning when the new rules and linguistic systems contradict the native ones. Cross-linguistic influence can be positive or negative, as learners transfer knowledge from their first language, facilitating learning when structures align or causing errors when they differ. This interactional phenomenon is explained by interlanguage theory (Selinker, 1972) and dynamic systems approaches (de Bot, 2008; Larsen-Freeman, 2013), which emphasize the evolving nature of bilingual development.

In addition to the cogent contribution of L1 features, other cognitive factors are involved in accounting for L2 learning and are related to independent factors such as cognitive mechanisms (e.g., long-term memory, short-term and working memory) and social environment (e.g., educational context and parental education; Bernhardt, 2000; Abbasian et al., 2020). The contribution of WM in L1 linguistic abilities is well established. It is required for different sub-processes such as remembering new information while reading, enabling inferences from what has been read, and the integration of long-term knowledge/new information (Daneman & Merikle, 1996). Given the relevance of WM in L1 learning, its contribution should be equally important in non-native language learning (Martin & Ellis, 2012; Rankin, 2017). However, WM is not a unitary construct, it is rather a combination of different components, whose contributions vary depending on age, task and the domain considered (e.g., Juffs & Harrington, 2011). This facilitates the individuation of two components involved in L2 learning: a passive storage component (i.e., memory capacity) and an active control component.

These two aspects are usually measured through different tasks, such as simple word/non word recall span to test (passive) capacity, and complex span tasks (e.g., dual tasks, where simultaneous maintenance and manipulation of information is needed, i.e., operation span task or updating task) to test the active control of, for example, the inhibition of one language.

Findings from developmental studies have supported the contribution of the passive WM component in accounting for L2 learning. In her seminal work, Service (1992) examined, in a three-year longitudinal study, Finnish children learning English as a second language at school. The results showed that measures of verbal short-term and phonological memory were the best predictors of English as a second-language acquisition three years later, even after controlling for general school achievement (see, for example, Georgiou & Giannakou, 2024; Hummel, 2021; Service & Kohonen, 1995).

Similarly, Masoura and Gathercole (1999) showed that the ability to repeat unfamiliar words at age four years predicts vocabulary at age five. In the same vein, Palladino and Ferrari (2008) showed a strong association between phonological memory and non-word learning in children; a less efficient phonological storage system (tested, for example, via syllable or letter deletion task, or non-word repetition) seems to characterize the memory profile of children with L2 learning difficulties. In sum, these studies illustrate a consistent developmental relation between the phonological processing of memory and L2 learning (e.g., vocabulary), independent of other factors such as visuospatial abilities.

Morra and Camba (2009) manipulated L1 and L2 word learning to examine the role of short-term/phonological memory, working memory capacity and long-term memory (previous vocabulary knowledge in children) in the two learning processes. They found that L2 word learning was mainly accounted for by short-term phonological memory, although some variance was also explained by the working memory-capacity measure (only for short words).

Nonetheless, alternative research avenues (e.g., Linck et al., 2014) have highlighted that complex span tasks are more reliable indicators of L2 proficiency than passive phonological tasks. These findings emphasize the importance of active executive functions over the passive storage aspects of WM. Specifically, Linck et al. (2014) reported a strong positive association between WM and L2 performance, with more substantial effects for executive control compared to simple storage. Further developmental evidence supporting the relevance of complex WM processes—such as updating, which involves retaining relevant information and discarding outdated content—was provided by Artuso and Palladino (2019), who found a direct relationship between WM updating abilities and L2 reading fluency.

In children with typical development, accurate non-word repetition has been shown to have a strong and specific connection to vocabulary development. This relationship was originally demonstrated in a longitudinal study involving children aged four to eight, who were assessed at four different time points on tasks measuring receptive vocabulary, non-word repetition, and nonverbal reasoning (Gathercole & Baddeley, 1989; Gathercole et al., 1992). At ages four, five, and six, there was a strong correlation between vocabulary knowledge and performance on non-word repetition tasks (Gathercole, 2006).

Comparably close and specific associations between non-word repetition and vocabulary knowledge have since been demonstrated in many other studies of the acquisition of vocabulary of both the native language (e.g., Gathercole et al., 1997; Michas & Henry, 1994) and foreign languages (Masoura & Gathercole, 1999; Gathercole & Masoura, 2005; Service, 1992; Service & Kohonen, 1995).

The relationship between non-word repetition and vocabulary knowledge appears to be most robust during the initial stages of language acquisition. This pattern of diminishing association has also been observed in more proficient second-language learners. Gathercole and Masoura (2005), for example, examined Greek-speaking children who had been exposed to English as a second language for an average of three years. Their results indicated a significant correlation between non-word repetition performance and receptive English vocabulary. However, when participants were asked to learn novel English lexical items paired with their Greek equivalents, non-word repetition ability no longer predicted performance. Instead, learning outcomes were more strongly associated with the learners’ pre-existing English vocabulary knowledge (see also, e.g., Hummel, 2021; Palladino & Ferrari, 2008).

The consistent and robust association between non-word repetition performance and language learning abilities prompt a deeper examination of what this task measures. A key limitation in non-word repetition lies in the need for precise phonological representations to accurately reproduce the auditory input. In this context, phonological storage is conceptualized in terms that closely align with the phonological short-term store described in Baddeley and Hitch’s (1974) model of the phonological loop. Linguistic input received through auditory channels is automatically encoded within this store, where the representations are vulnerable to rapid, time-based decay. This deterioration can be counteracted through subvocal rehearsal, a deliberate cognitive strategy that refreshes the memory trace by reactivating it. Rehearsal relies on internal articulatory mechanisms and typically does not emerge as a consistent strategy until around the age of seven (Gathercole & Hitch, 1993).

The phonological loop is commonly assessed using serial recall paradigms, in which verbal items are delivered at a uniform rate and must be reproduced instantly in the exact sequence of presentation. While the phonological loop is generally viewed as a temporary storage system separate from long-term lexical phonological knowledge, it does not function independently of more stable memory representations. Performance in immediate memory tasks is notably affected by the lexical properties of the items to be remembered. Specifically, serial recall tends to be more accurate for real words than for non-words (e.g., Hulme et al., 1991), and for high-frequency words compared to those that occur less frequently in the language (Hulme et al., 1997).

It is important to recognize that vocabulary acquisition cannot be explained by a single cognitive mechanism; rather, it involves a system composed of multiple interacting components (Morra & Camba, 2009; see also de Bot, 2008; Larsen-Freeman, 2013). To date, many studies have focused on individual factors to explain vocabulary learning such as the capacity of the phonological loop (e.g., Baddeley et al., 1998) or the precision of phonological representations (e.g., Bowey, 2001). Some research has suggested a dual-factor model, positing that both phonological memory and existing vocabulary knowledge contribute to the variance observed in word-learning outcomes (e.g., Gathercole & Masoura, 2005).

Baddeley’s multicomponent model and dual-factor accounts provide complementary perspectives on working memory. Baddeley conceptualizes WM as a modular system with domain-specific storage buffers coordinated by a central executive and an episodic buffer (Baddeley & Hitch, 1974; Baddeley, 2000), whereas dual-factor models distinguish between storage capacity and executive/attentional control as separable but correlated components (Engle et al., 1999; Kane et al., 2004). Both frameworks converge on the importance of domain-general executive processes, with the executive attention factor closely paralleling Baddeley’s central executive.

The work of Morra and Camba, however, highlights a more intricate pattern that aligns with Gathercole’s (2006) contemporary perspective, which views word learning as a multifaceted process involving phonological sensitivity that supports the formation of long-term representations that are accessed in the short term.

Morra and Camba (2009) propose that the latent predictor variables reflecting the cognitive system underlying learning encompass components of both long-term memory and working memory. Specifically, they argue that phonological memory can be further broken down into distinct elements, namely, phonological sensitivity (the efficiency of rehearsal processes), and the capacity of the memory system (M capacity). Phonological sensitivity emerges as a key predictor of vocabulary acquisition; however, when the target items are short and composed of familiar (native) phonological patterns, its predictive power diminishes. This is likely due to the relative ease with which children at this developmental stage can encode such sound patterns. In contrast, other types of non-words require the presence of clear, distinct speech sound representations and efficient processing mechanisms within long-term memory. For short non-words with familiar phonology, existing vocabulary knowledge becomes a much stronger predictor. This indicates that learners can leverage their current lexical knowledge when new items resemble words from their known language. In addition, Morra and Camba (2009) identify M capacity, conceptualized as a general attentional resource capable of activating a limited number of “schemes” or cognitive units, as a significant predictor for learning short non-words. It is worth noticing that with long-term memory, the authors refer to vocabulary knowledge.

Other authors support the interaction between short and long-term memory, always referred to as vocabulary knowledge, such as Gathercole et al. (1997), who hypothesized a reciprocal relationship between phonological memory and existing vocabulary knowledge. In the same vein, Jones et al. (2007) attempted to identify a computational model to link WM and long-term memory, to specify how new words are learned or how they are stored in long-term memory (see also Wang, 2024).

Based on this literature, we sought to investigate two main research questions: (1) What is the extent of improvement in L2 learning during the school year in a group of primary school students?; and (2) What is the effect of different memory systems on L2 learning?

We employed a six-month interval between measurements, a choice justified both theoretically and methodologically. This duration is long enough to capture meaningful developmental and cognitive changes, yet short enough to limit cohort effects and contextual variability. It balances sensitivity to change with participant retention, aligns with common practice in longitudinal cognitive and educational research, and provides sufficient temporal separation to model predictive or cross-lagged relationships, while maintaining conceptual continuity between waves.

## 3. Materials and Methods

### 3.1. Participants

Given that the present study aimed to explore whether long-term memory (delayed non-word recall) plays a predictive role in L2 vocabulary learning, we involved eight classes from 2nd and 3rd grade of a primary school in southern Italy. As shown in Table 1, the total sample was composed of 82 primary Italian students aged between 6.3 and 9.6 years (M_Age_ = 7.8 years; SD = 0.64; 43 females, 52%). Participation was dependent on written informed consent from each student’s parents and/or legal caretakers. All children were required to be either native Italian speakers or sufficiently fluent in Italian. We initially involved in the activities all participants who had neurological, psychological, or behavioral problems, and who had parental consent. However, these students were excluded from the data analysis.

### 3.2. Procedure

Children were assessed twice, always during school time, on the same measures: first in November 2021 (Time 0—T0) and again, 6 months later, in May 2022 (Time 1—T1). Each student was tested individually in a quiet room close to their own class. The test had an average duration of 30 min. The study was carried out according to the ethical guidelines of the Italian Association of Psychology (AIP); for each participant, informed consent was obtained in accordance with the ethical standard of the Declaration of Helsinki (1975, as revised in 2008). The study was approved by the Ethics Committee of the Psychology section of University of Foggia (approval of project number 34/19, Year 2019). All measures involved are listed below.

### 3.3. Materials

Immediate non-word recall: To assess students’ memory span, a list of ten disyllabic non-words was used as the first test (Marinelli et al., 2016). The same list was given to second- and third-grade students, in the same order of presentation, namely: “zila”; “muci”; “esfi”; “siba”; “libo”; “bipo”; “fipo”; “mafe”; “gomi”; “urse”. The researcher presented the list, encouraging students to pay close attention, as they would have to repeat as many non-words as they could remember. The task involved five alternating repetitions: between one repetition by the researcher and the next, the student attempted to repeat the memorized words. The score corresponded to the total number of non-words correctly repeated in each phase.

*Digit Span Forward and Backward:* Short-term verbal memory was measured using both forward and backward digit span tests in the L1 version (Orsini et al., 2012). Each test included 21 numerical sequences, with lengths ranging from a minimum of 3 numbers (forward) and 2 numbers (backward), up to a maximum of 9 (forward) and 8 (backward), excluding zero. The sequences were organized according to length and mnemonic complexity. Again, the same sequences were used for both classes. In the forward version, the student was required to repeat the sequence in the same order in which it was read by the researcher; in the backward version, they were required to repeat it in reverse (for example, “3-8-6” became “6-8-3”). The test stopped after three consecutive errors in the same length of the sequence. However, if one or two consecutive errors was followed by a correct answer, the task continued.

*Delayed Non-Word Recall:* In addition to short-term memory, we also assessed long-term memory (specifically, episodic memory) using delayed non-word recall. During the time between the initial test and this one (approximately 10 min), students were engaged in other activities (such as the numerical span test) to avoid constant review of the non-words. Subsequently, students were asked to recall all the non-words learned in the first exercise. The test had no time limit and ended when the student declared they could not remember anything else. One point was awarded for each correctly recalled non-word, regardless of any repetitions (for example, if a student repeated the same correct word, such as “FIPO,” twice, they still only obtained one point).

*L2 performance*: To evaluate the L2 vocabulary performance (specifically, English), we introduced students to three different short tasks, administered individually: vocabulary entry; vocabulary output; and sentence building (Palladino et al., 2025). The tasks had different items for the 2nd and 3rd grades, created according to national indications for the school grade curriculum. For the vocabulary entry, we introduced the participants to six stimuli: school supplies for 2nd grade, and clothing for 3rd grade. The researcher said, out loud, a specific term in English, and the student was required to point to the corresponding image. For the vocabulary output, the request was overturned. Introducing the student to six new stimuli (animals for 2nd grade, rooms of the house for 3rd grade), the researcher pointed to a stimulus; the student was required to say the specific term out loud. The score for both tasks was calculated from a minimum of zero to a maximum of six, assigning one point for each correct answer, and zero points for each incorrect answer. For the sentence building, we introduced two different topics for each grade. In the first, we asked students to produce a simple Italian phrase in English (e.g., *“Come si chiede ad un bambino chi è il suo cantante preferito, in inglese?”*). In the second, we introduced a concept in Italian, translated it ourselves into English, and asked students to reply in English (e.g., *“mi chiedono a che ora comincio la scuola. When is your class starting?”*). The sentence building score was calculated as the number of words that made up the grammatically correct sentence form of each answer. Any additional words (e.g., good morning; please; etc.) were not taken into consideration. The final score consisted of the sum of the correct answers in the vocabulary entry (maximum 6), in the vocabulary output (maximum 6) and in the sentence building (maximum 10 for the 2nd grade, and 11 for the 3rd grade), ranging from 0 to 22 or to 23.

## 4. Results

### 4.1. Comparing Performance at T0 and at T1

First, descriptive statistics on interest variables were computed at T0 and T1. Table 2 reports the mean performance at both timepoints in the whole sample (N = 82). To assess any potential variation in these variables across the time (T0 vs. T1), a series of paired *t*-tests were performed. No statistical difference was found when comparing digit span forward, t (81) = −1.11, *p* > .05, digit span backward, t (81) = 1.25, *p* > .05, and delayed non-word recall, t (81) = −1.24, *p* < .001, at T0 and T1. On the contrary, a significant increase in the immediate non-word recall, t (81) = −5.08, *p* < .001, and in L2 performance, t (81) = −5.57, *p* < .001, were detected. More deeply analyzing the emerged difference in the L2 performance, it was highlighted that students ameliorated their performance from T0 to T1 in all the L2 tasks, with the exception of vocabulary output, t (81) = −0.84, *p* > .05. In particular, a significant difference was found in vocabulary entry, t (81) = −4.09, *p* < .001, in the sentence building task—English production (EP), t (81) = −2.43, *p* < .05, and in the sentence building task—English reply (ER), t (81) = −3.91, *p* < .001).

### 4.2. Gender and Grade Effect on L2 Performance

To test the presence of gender and grade difference in L2 performance, we conducted a two-way ANOVA to test the effect of gender (f, m) and grade (2nd, 3rd) on L2 performance at T0.

Results highlighted a significant main effect of gender, *F*(1, 78) = 4.71, *p* = .33, *η*^2^ = 0.57, with female students reporting a better L2 performance (M = 6.58, S.E. = 0.44) than male students (M = 5.17, S.E. = 0.47) at the beginning of the school year (T0). In addition, a significant interaction effect, *F*(1, 78) = 5.07, *p* = .027, *η*^2^ = 0.061, was detected. Pairwise comparisons revealed that, while the 2nd grade students’ L2 performance was not statistically different between female (M = 5.34, S.E. = 0.57) and male (M = 5.39, S.E. = 0.76), for 3rd grade students, the difference between female (M = 7.83, S.E. = 0.67) and male (M = 4.96, S.E. = 0.57) L2 performance was statistically significant (mean difference = 2.87, S.E. = 0.88, *p* = .002). These results seem to suggest that, at the beginning of the school year (T0), female 3rd grade students showed an a priori advantage in L2 performance, compared to all the other students.

**Figure 1:**
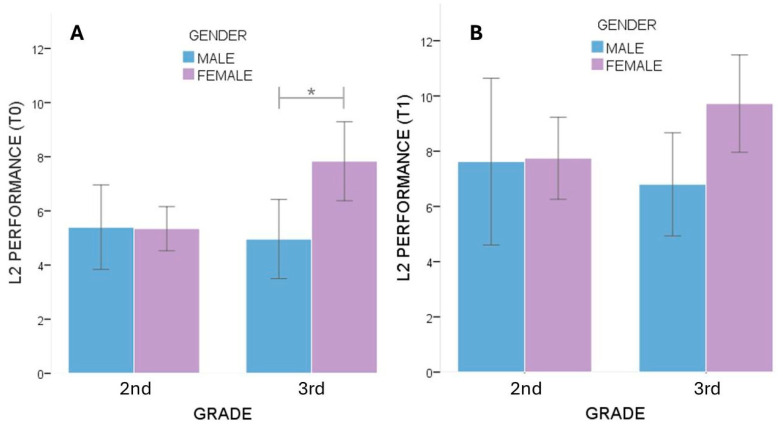
(**A**,**B**) L2 performance at T0 and T1 by gender and grade. * *p* < .05.

The same two-way ANOVA was conducted on L2 performance at the end of the school year (T1). Neither the main effect of gender, *F*(1, 78) = 2.53, *p* = .11, nor the main effect of grade, *F*(1, 78) = 0.36, *p* = .54, nor the interaction effect *F*(1, 78) = 2.15, *p* = .14, were found to be significant. These results indicate that, at the end of the school year (T1), the female 3rd grade students may have lost their initial advantage (Figure 1A,B).

As a last step, a mixed ANOVA on L2 performance, with time (T0, T1) as the within-factor and gender (f, m) and grade (2nd, 3rd) as the between-factors, was conducted. A significant main effect of time was found, *F*(1, 78) = 28.44, *p* < .001, *η*^2^ = 0.267, with students reporting a better L2 performance at T1 (M = 7.97, S.E. = 0.47) compared to T0 (M = 5.88, S.E. = 0.32). In addition, the main effect of gender, *F*(1, 78) = 28.44, *p* < .001, *η*^2^ = 0.267, and gender by grade interaction effect, *F*(1, 78) = 28.44, *p* < .001, *η*^2^ = 0.267, were confirmed as significant. All the pairwise comparisons by gender are reported in Table 3.

### 4.3. Predicting L2 Performance Variation

**Figure 2:**
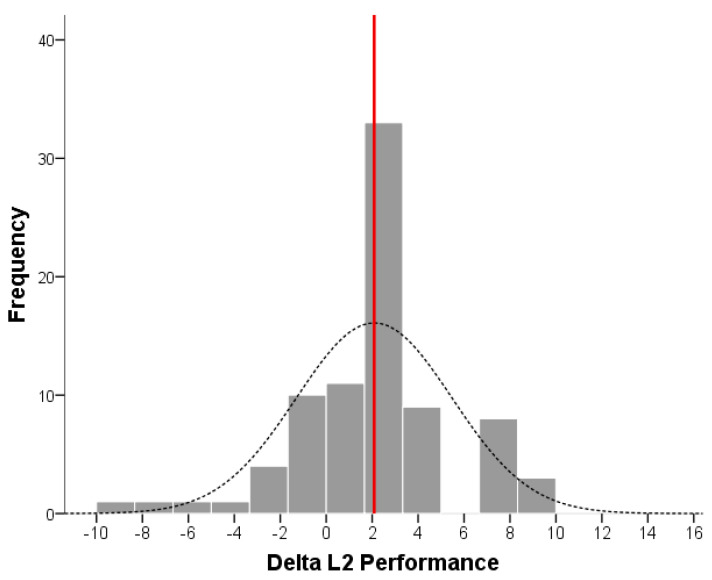
Distribution of delta L2 performance. The red vertical line refers to the median position (Median = 2.1).

To address whether any of the assessed variables at T0 were significantly associated with the variation in L2 performance, a delta parameter (L2 performance at T1 − L2 performance at T0) was computed. A delta value > 0 indicates progress in the L2 performance across time. Considering the whole sample (see Figure 2), the delta parameter ranged from −9 to 10 (M = 2.09, S.D. = 3.38, skewness = 0.44, kurtosis = 1.77). A delta value < 0, indicating a worsening of L2 performance from T0 to T1, was observed in 17.1% of the sample, while 4.9% of the sample maintained a stable performance across time (delta = 0) and 78% of participants showed an increased performance (delta > 0). A significant positive correlation was found between delta L2’s performance and the delayed non-word recall performance at T0 (r = 0.203, *p* < .05, Table S1 Supplementary Materials). To assess if memory performance at T0 predicts variation in L2 performance from T0 to T1, a multiple regression on delta L2 performance was run, entering memory variables at T0 (immediate non-word recall, digit span forward, digit span backward, delayed non-word recall) as predictors. The full results are reported in Table 4. While the model was not significant, the results highlight a significant effect of delayed non-word recall at T0 (t = 2.04, *p* < .05) in predicting progress in L2 performance across time.

### 4.4. Gender and Grade Differences in Delayed Non-Word Recall at T0

**Figure 3:**
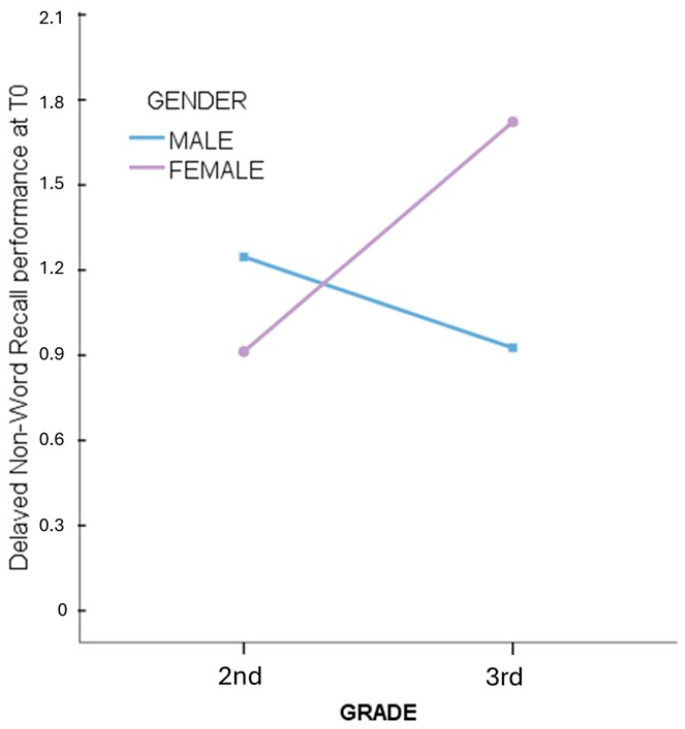
Gender- and grade-based differences in delayed non-word recall at T0.

A two-way ANOVA was conducted to address the effect of gender and grade on the delayed non-word recall performance at T0. A significant interaction effect was detected, *F*(1, 78) = 4.55, *p* < .05, η^2^ = 0.055. Table 5A,B report post hoc comparisons by gender and by grade, respectively. The results highlighted that while 2nd grade male and female students showed the same delayed non-word recall performance at T0, when considering 3rd grade students, the female students reported a significantly higher performance (M = 1.72, S.E. = 0.27) than male students (M = 0.926, S.E. = 0.23). When comparing same-gendered 2nd and 3rd grade students, only female students presented a better performance in 3rd grade compared to 2nd grade (M = 0.913, S.E. = 0.23) female participants. On the contrary, the delayed non-word recall performance of 2nd (M = 1.24, S.E. = 0.31) and 3rd grade male students did not differ (Figure 3). Taken together, the results seem to indicate that progressing from the 2nd to the 3rd grade may be associated with a relevant improvement in long-term memory for females but not for male children. Based on the observation that the dynamics of memory skill development and refinement may progress differently based on gender, and that this, in turn, may exert different influence on L2 performance variation, a moderation model was run to test the effect of delayed non-word recall (X) on delta L2 performance (Y), with gender as the moderator (M).

**Figure 4:**
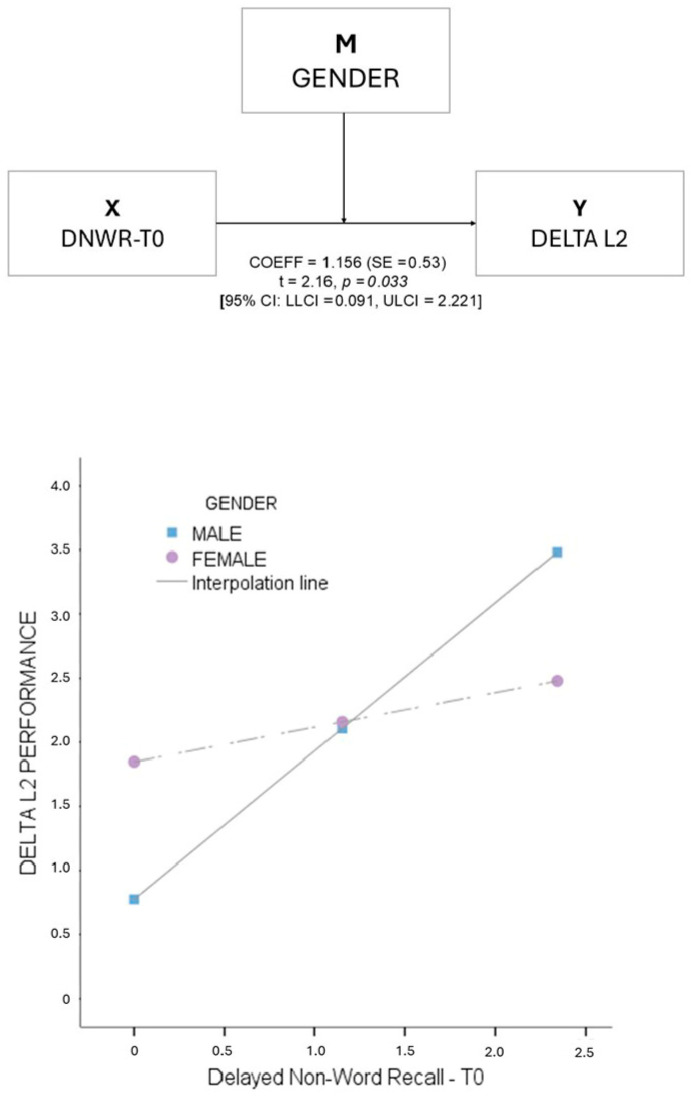
Moderation model and conditional effect of delayed non-word recall on delta L2 performance in male and female students.

The effect of delayed non-word recall on delta L2 performance was found to be significant [coefficient = 1.156, S.E. = 0.534, t = 2.164, *p* < .05, LLCI = 0.0912, ULCI = 2.221], while this was not true for the delayed non-word recall x gender interaction effect (Figure 4).

An analysis of the conditional effect of X on Y at the values of the moderator showed that the effect of delayed non-word recall on delta L2 performance was significant when considering the male participants, but not the female participants [coefficient = 0.270, S.E. = 0.387, t = 0.698, *p* > .05, LLCI = −0.500, ULCI = 1.040].

### 4.5. Primacy Effect

The recall frequencies for each non-word during the delayed non-word recall task, with ZILA (the first target non-word) emerging as the most frequently recalled, are reported in Table S2 (see Supplementary Materials).

Observing a potential primacy effect involving the first two non-words (ZILA and MUCI), we conducted a series of log-linear regressions to evaluate the significance of recall differences across all non-words (Model 1), and with each of the first two non-words serving as the reference level in separate analyses (Models 2 and 3, respectively). As shown in Table S3 (see Supplementary Materials), ZILA is the most frequently recalled word, significantly more than almost all the others, except for MUCI, confirming the primacy effect. FIPO and ESFI were among the least recalled, significantly less than both ZILA and MUCI.

## 5. Discussion

Learning L2 English as a second-language vocabulary requires multiple interacting memory components (Morra & Camba, 2009). The role of the phonological loop (e.g., Baddeley et al., 1998) and the precision of phonological representations (e.g., Bowey, 2001) as well as the relevance of the existing vocabulary knowledge appear to have been consistently demonstrated (e.g., Gathercole & Masoura, 2005). However, in explaining word learning as a process that supports the formation of long-term representations, a connection between short- and long-term memory is missing.

First, we examined the development of L2 learning, expecting an improvement in performance at increasing ages—showing that as age increases, L2 performance ameliorates (e.g., Gathercole et al., 1992, 1997). With respect to gender, we observed higher L2 performances in females compared to males at T0 compared, aligning with prior research suggesting an early female advantage in L2 acquisition timing (e.g., Brouwer et al., 2021; Kheder & Rouabhia, 2023). However, this advantage was no longer significant after six months (T1), as male performance reached parity with that of their female peers. A notable finding is that phonological memory predicted L2 improvement over time exclusively in male students. This suggests a potential divergence in cognitive resource utilization as, while males appear to rely heavily on a phonological route for vocabulary growth, female students may be recruiting alternative cognitive resources that were not the primary focus of the current study. While some studies in the literature suggest that environmental or school-related factors could differentially impact gender-based learning trajectories (Martinot et al., 2025), our current data do not provide direct evidence for such external influences. Consequently, the narrowing of the gender gap over six months should be interpreted as a developmental shift in the relative importance of phonological memory across groups, rather than a definitive statement on pedagogical or environmental causality. Overall, research on gender differences suggests that females often outperform males in verbal fluency and memory tasks during early development (Kaushanskaya et al., 2013). This may be attributed to different neurological maturation rates, with some studies suggesting an advantage for females of up to one year (Khan et al., 2024). Our study may extend this investigation by contributing to an understanding of how this initial advantage interacts with memory consolidation efficiency in time.

In the present study, we also tested the role of different memory components—short- and long-term—to better understand their role in second-language vocabulary learning in primary school students through a longitudinal study (two timepoints over six months). The results showed significant correlation between short-term memory (forward, but not backward span), and long-term memory (delayed non-word recall) but not phonological short-term memory (immediate non-word repetition) measured at T0 with L2 vocabulary learning at T1.

To establish which of the measures best predict L2 vocabulary acquisition, a series of regression analyses was conducted that showed the key role of delayed non-word recall as a variable that accounts for L2 learning change, even if only with a moderate intensity effect (see Table 4). In addition, a stronger role for long-term memory representation, here exemplified by delayed non-word recall performance, was shown in the analyses of single item recall. As shown in Tables S2 and S3, a noteworthy primacy effect was observed, which is the first demonstration of an initial long-term consolidation of to-be-recalled non-words. While the literature seems to agree on the role of short-term phonological memory (e.g., Gathercole et al., 1992, 1997) and WM (Juffs & Harrington, 2011), the present finding represents a challenge for the literature, adding a new memory component (first stages of long-term memory consolidation), which is likely to impact second-language acquisition. Some research evidence with respect to the role of long-term components has been obtained from computational models (e.g., Jones et al., 2007) that attempt to link WM and long-term memory, in order to specify how new words are learned or how they are stored in long-term memory. Additional strength to the role of long-term memory components was indicated in the non-word position analysis, which supported a primacy effect, generally known as a strong marker of long-term memory (e.g., Page & Norris, 1998). The observed primacy effect in delayed non-word recall may indeed offer a window into the initial stages of long-term lexical consolidation. According to the maintenance rehearsal hypothesis, items at the beginning of a sequence receive a disproportionate amount of cognitive resources and covert rehearsal, facilitating their transfer from the phonological loop to long-term episodic memory (Baddeley et al., 2019). In the context of L2 acquisition, this advantage in the memory trace is theoretically significant, as words that benefit from primacy are more likely to achieve the phonological stability required for permanent lexical integration. By linking these item-level recall patterns to L2 outcomes, we suggest that the primacy effect acts as a cognitive filter, determining which novel phonetic strings are prioritized for the structural remodeling of the mental lexicon, thereby informing theories of how transient acoustic signals become stable linguistic representations.

Based on this, the findings of this study may offer significant pedagogical insights for L2 vocabulary instruction in primary education. Since delayed non-word recall proved to be a superior predictor compared to immediate repetition, instruction should shift focus from rote immediate mimicry to activities that promote a deeper encoding. Practically, the observed primacy effect may also indicate that teachers should introduce the most critical or difficult vocabulary at the very beginning of a lesson, when cognitive resources for long-term consolidation are at their peak. Furthermore, because the transition from short-term to long-term representation is a critical point for many learners, incorporating spaced retrieval practices may help to reach this aim. This involves revisiting new words at increasing intervals to strengthen the initial long-term memory traces.

## 6. Conclusions

In sum, in this longitudinal study we originally showed the role of long-term memory as a key factor in learning a second language. The primary contribution of this study is the demonstration that L2 vocabulary growth is predicted more accurately by initial consolidation efficiency (delayed non-word recall) than by immediate short-term capacity. This suggests that L2 acquisition models should explicitly incorporate long-term anchoring mechanisms as a critical factor in successful L2 vocabulary acquisition. Our results demonstrate that while age and gender influence initial performance, the major prompt of L2 vocabulary growth may be represented by the initial consolidation of long-term representations, rather than simple immediate phonological repetition. The presence of a significant primacy effect provides empirical evidence for this “missing link” between short-term and long-term memory systems, challenging traditional models that rely solely on the phonological loop.

In addition, we contributed to the literature on age and gender effects on language learning by showing that females show an advantage over male at the beginning of L2 learning that is lost over the course of the school year. This finding deserves further attention and exploration and might be related to the detrimental effects of school on female performance, as has already been demonstrated for STEM school subjects (e.g., Martinot et al., 2025).

Despite the longitudinal design, this study is not without limitations. First, the sample size, while sufficient for the primary analyses, may have limited the statistical power needed to fully tease out the complex gender-related interactions over time. Second, the study focused exclusively on vocabulary; therefore, the results may not generalize to the acquisition of other L2 components. Lastly, the six-month interval between T0 and T1 provides a picture of development; however, a longer multi-year study would be required to see if these memory predictors remain stable as L2 proficiency increases.

## Figures and Tables

**Table 1:** Descriptive statistics of the sample. N = Sample size; M_Age_ = Mean; SD = Standard deviation.

Gender	School Grade	N	M_Age_ (SD)
Male	2nd	14	7.45 (0.81)
	3rd	25	8.04 (0.59)
Female	2nd	25	7.54 (0.49)
	3rd	18	8.22 (0.44)

**Table 2:** Mean values of interest variables in the whole sample at T0 and T1. Standard deviation in brackets. Mean difference significant at ** *p* < .001; * *p* < .05.

Variable	T0	T1	Mean Difference
Immediate Non-Word Recall	6.8 (3.5)	9.7 (5.7)	2.9 **
Digit Span Forward	4.2 (0.62)	4.3 (0.62)	0.1
Digit Span Backward	2.7 (0.66)	2.6 (0.65)	−0.1
Delayed Non-Word Recall	1.1 (1.2)	1.2 (1.2)	0.1
L2 Performance	5.8 (3.1)	7.9 (4.2)	2.1 **
Vocabulary Entry	2.2 (1.5)	3.1 (1.5)	−0.9 **
Vocabulary Output	1.8 (1.4)	1.9 (1.3)	−0.1
Sentence Building Task EP	1.0 (1.3)	1.5 (1.6)	−0.4 *
Sentence Building Task ER	0.7 (1.1)	1.3 (1.2)	−0.6 **

**Table 3:** Gender pairwise comparisons.

Grade	Time	Gender	Gender	Mean Difference	Standard Error	*p* Value	LLCI 95%	ULCI 95%
2nd	T0	M	F	0.05	0.95	.956	−1.84	1.95
	T1	M	F	−0.11	1.40	.933	−2.90	2.66
3rd	T0	M	F	−2.87	0.88	.002	−4.63	−1.11
	T1	M	F	−2.92	1.29	.027	−5.50	−0.34

**Table 4:** Regression model.

Model	Non-Standardized Coefficients		Standardized Coefficients	t	*p*-Value
	*T*	S.E.	*Β*		
Constant	−1.04	2.78		−0.376	.708
Immediate Non-Word Recall	−0.152	0.127	−0.160	−1.19	.235
Digit Span forward	0.386	0.630	0.071	0.614	.541
Digit Span backward	0.620	0.568	0.122	1.09	.278
Delayed Non-Word Recall	0.755	0.370	0.265	2.04	.045
			*F*(4, 77) = 1.53, *p* > .05 *R*^2^ = 0.074		

**Table 5:** (**A**) Post-hoc comparisons by gender (**B**) Post-hoc comparisons by grade.

**(A)**							
**Grade**	**Gender**	**Gender**	**Mean** **Difference**	**Standard** **Error**	***p* Value**	**LLCI 95%**	**ULCI 95%**
2nd	Male	Female	0.334	0.388	.393	−0.440	1.108
3rd	Male	Female	−0.796	0.360	.030	−1.513	−0.078
(**B**)							
**Gender**	**Grade**	**Grade**	**Mean** **Difference**	**Standard** **Error**	***p* Value**	**LLCI 95%**	**ULCI 95%**
Male	2nd	3rd	0.321	0.389	.412	−0.453	1.095
Female	2nd	3rd	−0.809	0.360	.027	−1.526	−0.093

## Data Availability

The data that support the findings of this study are available from the corresponding author upon request.

## Supplementary Materials

The following supporting information can be downloaded at: https://www.mdpi.com/article/10.3390/jintelligence14060092/s1. Table S1. Correlations between interest variables in the whole sample at T0 and T1. Significant at ** *p* < .001; * *p* < .05. Table S2. Estimated Marginal Means recall frequencies for each nonword during the Delayed Nonword Recall task. Table S3. Comparison of Linear Log-Regression Estimates.

## Abbreviations

The following abbreviations are used in this manuscript:

AIP	Associazione Italiana di Psicologia
ANOVA	Analysis of variance
EP	English production
ER	English reply
f	Female
L1	Native language
L2	Second language
M	Mean
m	Male
N	Sample size
S.E.	Standard error
SD	Standard deviation
T0	Time 0
T1	Time 1
WM	Working memory
