# Supplementary material for: How Memory Shapes Second-Language Vocabulary Acquisition in Primary School Learners
Source: J Intell. 2026 Jun 1;14(6):92. doi: 10.3390/jintelligence14060092 (PMC13302240; doi:10.3390/jintelligence14060092)
Supplement: Supplementary file 1 [file jintelligence-14-00092-s001.zip › jintelligence-4082992-supplementary.pdf]

Supplementary Material

Correlations

Pearson correlations analysis between the interest variables at T0 and T1 was conducted. A full prospect is reported in Table 3. Of particular interest are the emerged significant positive correlations between the Digit Span forward performance at T0 and the L2 performance at T1 ($r = .370$, $p < .001$) and between the Delayed Non-Word Recall Performance at T0 and the L2 performance at T1 ($r = .228$, $p < .05$). Nevertheless, these significant correlations refer to static L2 performance at T1 and do not address the (expected) variation of the L2 performance from T0 to T1.

*Table S1***.** Correlations between interest variables in the whole sample at T0 and T1. Significant at **p < .001; * p < .05.

**Variables**	**1.**	**2.**	**3.**	**4.**	**5.**	**6.**	**7.**	**8.**	**9.**	**10.**
**1.** T0 - Immediate Non-Word Recall	1									
**2.** T0 - Digit Span forward	.294**	1								
**3.** T0 - Digit Span backward	.130	.165	1							
**4.** T0 - Delayed Non-Word Recall	.533**	.212*	.063	1						
**5.** T0 - L2 Performance	.215*	.409**	.107	.094	1					
**6.** T1 - Immediate Non-Word Recall	.489**	.275**	.089	.396**	.331**	1				
**7.** T1 - Digit Span forward	.294**	.988**	.165	.212*	.409**	.275**	1			
**8.** T1 - Digit Span backward	.142	.145	.986**	.051	.121	.058	.145	1		
**9.** T1 - Delayed Non-Word Recall	.522**	.222*	.082	.987**	.087	.409**	.222*	.055	1	
**10.** T1 - L2 Performance	.168	.370**	.179	.228*	.612**	.520**	.370**	.169	.233*	1
**11.** Delta L2 Performance	.019	.101	.130	.203*						

Table S2. Estimated Marginal Means recall frequencies for each nonword during the Delayed Nonword Recall task.

Non-Words	95% Confidence Interval		Lower	Upper
	Frequencies	SE		
ZILA	22.00	4.69	14.49	33.41
MUCI	13.00	3.61	7.55	22.39
LIBO	7.00	2.65	3.34	14.68
ESFI	2.00	1.41	0.50	8.00

SIBA	7.00	2.65	3.34	14.68
MAFE	5.00	2.24	2.08	12.01
BIPO	5.00	2.24	2.08	12.01
GOMI	9.00	3.00	4.68	17.30
FIPO	1.00	1.00	0.14	7.10
URSE	6.00	2.45	2.70	13.36

Table S3. Comparison of Linear Log-Regression Estimates.

Predictor	Model 1	Model 2 (ZILA)				Model 3 (MUCI)			
	(All Non-words)	Estimates	SE	Z	*p*-value	Estimates	SE	Z	*p*-value
ZILA (intercept for model 2)		3.09	0.21	14.50	<.001	0.53	0.35	1.50	0.133
MUCI (intercept for model 3)		-0.53	0.35	-1.50	0.133	2.56	0.28	9.25	<.001
LIBO		-1.15	0.43	-2.64	0.008	-0.62	0.47	-1.32	0.187
ESFI		-2.40	0.74	-3.25	0.001	-1.87	0.76	-2.46	0.014
SIBA		-1.15	0.43	-2.64	0.008	-0.62	0.47	-1.32	0.187
MAFE		-1.48	0.50	-2.99	0.003	-0.96	0.53	-1.82	0.069
BIPO		-1.48	0.50	-2.99	0.003	-0.96	0.53	-1.82	0.069
GOMI		-0.89	0.40	-2.26	0.024	-0.37	0.43	-0.85	0.396
FIPO		-3.09	1.02	-3.02	0.003	-2.56	1.04	-2.47	0.013
URSE		-1.30	0.46	-2.82	0.005	-0.77	0.49	-1.57	0.117
Model Fit Measures									
Deviance	0.00								
AIC	56.25								
BIC	59.28								
R^2	1.00								
χ^2	38.84***								
Df	9								

Note. *p<.05; **p<.01; ***p<.001.
